# Supplementary material for: AffyMAPSDetector: a software tool to characterize Affymetrix GeneChip™ expression arrays with respect to SNPs
Source: BMC Bioinformatics. 2007 Jul 30;8:276. doi: 10.1186/1471-2105-8-276 (PMC1959249; doi:10.1186/1471-2105-8-276)
Supplement: Additional file 13 — AffyMAPSDetector v1 source code. AffyMAPSDetector source code. [file 1471-2105-8-276-S13.zip › AffyMAPSDetector_v1_src/doc/affychip/snpdetector/resources/ResourceLoader.html]

ResourceLoader


|  |  |  |  |
| --- | --- | --- | --- |
| **Overview** \| **Package** \| **Class** \| **Use** \| **Tree** \| **Deprecated** \| **Index** \| **Help** | | |  |
| **PREV CLASS**   NEXT CLASS | **FRAMES**    **NO FRAMES**     **All Classes** |  |  |
| SUMMARY: NESTED \| FIELD \| CONSTR \| METHOD | DETAIL: FIELD \| CONSTR \| METHOD |  |  |


---


## affychip.snpdetector.resources Class ResourceLoader

```
java.lang.Object
  affychip.snpdetector.resources.ResourceLoader
```

---

public class **ResourceLoader** extends java.lang.Object

Title: Probe SNP Detector

Description: Affymetrix Gene-Probe SNP Detection

This provides uniform and easy way to load application specific resources.
For example: logging config file, images etc.

Copyright: Copyright (c) 2004

Company: George Mason University

**Version:**
:   1.1

**Author:**
:   Sunita Kumari

---

|  |  |
| --- | --- |
| **Field Summary** | |
| `static org.apache.log4j.Logger` | `logger` |


**Constructor Summary**

`ResourceLoader()`


|  |  |
| --- | --- |
| **Method Summary** | |
| `static void` | `loadResources()` |

**Methods inherited from class java.lang.Object**

`clone, equals, finalize, getClass, hashCode, notify, notifyAll, toString, wait, wait, wait`

**Field Detail**

### logger

```
public static org.apache.log4j.Logger logger
```


**Constructor Detail**

### ResourceLoader

```
public ResourceLoader()
```


**Method Detail**

### loadResources

```
public static void loadResources()
```


---


|  |  |  |  |
| --- | --- | --- | --- |
| **Overview** \| **Package** \| **Class** \| **Use** \| **Tree** \| **Deprecated** \| **Index** \| **Help** | | |  |
| **PREV CLASS**   NEXT CLASS | **FRAMES**    **NO FRAMES**     **All Classes** |  |  |
| SUMMARY: NESTED \| FIELD \| CONSTR \| METHOD | DETAIL: FIELD \| CONSTR \| METHOD |  |  |


---
